# Quality Food Products as a Tourist Attraction in the Province of Córdoba (Spain)

**DOI:** 10.3390/ijerph191912754

**Published:** 2022-10-05

**Authors:** Mª Genoveva Dancausa Millán, Mª Genoveva Millán Vázquez de la Torre

**Affiliations:** 1 Department of Statistics, Córdoba University, 14071 Córdoba, Spain; 2 Department of Quantitative Methods, Universidad Loyola Andalucía, 14004 Córdoba, Spain

**Keywords:** gastronomic routes, Iberian ham, olive oil, wine, Córdoba, ARIMA, food, gastronomic tourism

## Abstract

Traveling to learn about the gastronomy of a destination is becoming increasingly important among tourists, especially in the wake of the pandemic. Quality foods endorsed by protected designations of origin (PDOs) are increasingly in demand, as are experiences related to their production processes. In this study, the seven PDOs in the province of Córdoba (Spain) are analyzed. These PDOs produce olive oil, wine or ham. A field study was performed, whereby 315 gastronomic tourists who visited a gastronomic route or a PDO in Córdoba were surveyed. The objective was to characterize the profile of visiting tourists and to anticipate future demand using ARIMA models. The results indicate that the growth in gastronomic tourism in Córdoba is lower than that in the wider region, and that there are no significant differences among the different profiles (oil tourist, enotourist and ham tourists) due in part to the fact that most tourists travel from nearby regions. The novelty of this study is that three products are analyzed, and strategies are proposed to deseasonalize this type of tourism, for example, by creating a gastronomic brand that represents Córdoba and selling products under that brand (especially in international markets), by highlighting raw materials and prepared dishes and by making gastronomy a complement to heritage tourism in the city and rural tourism in the province.

## 1. Introduction

The gastronomy of a region is part of its culture and identifies food products with the territory [1,2], constituting both a material heritage formed by a raw material and agri-food products, such as cheese, wine, oil, etc., ham, etc., and an intangible heritage shaped by the recipes with which food dishes are prepared, culinary traditions, etc. [3]. This heritage is being increasingly appreciated by people who taste these foods, constituting potential resources that serve as a basis for the growing development of gastronomy because geographical and cultural diversity provides a great variety of foods and ways of preparing them [4], with food being considered an essential good as well as an integral part of the social and cultural heritage [5] that can be exploited not only from the perspective of food but also as a tourist product.

Improvements in gastronomic resources are providing new opportunities to many territories, especially rural ones; gastronomy is becoming not only an identity for the residents of an area but also an attraction for potential tourists who want to learn about it.

Since the beginning of the 21st century, people have become more concerned with having a balanced and healthy diet and spending their leisure time visiting destinations where food is of high quality to learn about how it is produced, especially foods belonging to the Mediterranean diet. The Mediterranean diet is based on various nutrients, including *fats* such as olive oil, that yield vitamins and antioxidants that are very beneficial to human health [6,7]; *meats,* which provide protein, vitamins and minerals (including, among other cured meats, Iberian ham); *fruits*, which are a source of vitamins, fiber, water and minerals; and *vegetables,* which provide fiber, vitamins, minerals and carbohydrates. The great variety of products that comprise the Mediterranean diet are attracting an increasing number of tourists who want to learn about it, with gastronomy being one of the main reasons to visit cultural destinations such as Italy [8] and Greece [9]. This diet has a lower environmental impact because it includes many plant-derived and fewer animal products [10], and it is better for human health [11,12].

Of all the foods mentioned above, in this study, we will analyze Iberian ham, wine and olive oil as elements of Córdoba gastronomy, and from a tourist attraction perspective, to determine the factors that characterize the gastronomic tourist and to estimate potential demand.

Andalusia, an region located in the south of Spain, has a rich gastronomy comprising dishes whose recipes date back more than a thousand years, such as octopus casserole or eggplant almodrote, whose origin stems from the region’s Jewish heritage. During the 800 years of coexistence of Jewish and Muslim people in the Iberian Peninsula, there are dishes in which Judo–Christian–Arab culinary traditions have been mixed, creating a fusion of gastronomic elements where culinary traditions are combined, giving rise to the current Andalusian cuisine.

The methods of preparing dishes and the quality of the food elements that constitute typical dishes at a destination are now generating tourism, which is gaining more relevance every day in Andalusia and is becoming the main reason for tourists to visit certain areas of the region.

Córdoba, one of the eight provinces that are part of the Andalusia region, has a continental Mediterranean climate, with temperatures ranging between 9.2 °C in January and 36 °C in July and August. Rainfall varies between 400 to 600 mm per year, concentrated from October to April. Its vegetation is characterized by Mediterranean forest, in which trees of different oak ecotypes and other autochthonous herbaceous plants predominate, forming a landscape called *dehesa*, which is defined by its three functional zones: pasture, arable and mountain. The morphology of the terrain is characterized by shallow, acidic soils with an abundance of granites, gneisses or slates. Such morphology makes these lands agriculturally very unproductive. However, rain-fed crops grow here, as do the olive and the holm oak, whose fruit, the acorn, is the main food of the Iberian pig. The region’s capital includes four World Heritage declaration sites and is a heritage city. Cultural tourism is one of the main drivers of its economy, which has been severely affected by the COVID-19 pandemic; however, the province relies mainly on agriculture and livestock, with tourist activity representing complementary income to the fundamental activities of the primary sector.

Rural tourism, active tourism, cultural tourism and gastronomic tourism are among the main tourist activities that an individual can engage in when visiting the province.

Every year, thousands of people visit the province to taste typical Córdoba dishes, such as *flamenquín*, *rabo de toro* (braised oxtails), and *salmorejo*, with recipes from Arab and Jewish heritage that are made with top-quality raw materials, such as olive oil, ham or wine endorsed by protected designations of origin (PDOs) that certify the quality of these products [13].

However, despite having these raw materials, tourist demand is still low compared to that for other gastronomic destinations. What is happening with gastronomic tourism in Córdoba? Why is it still in the takeoff phase despite having seven PDOs and two restaurants that have received Michelin stars (NOOR Restaurant, 2 stars; Choco restaurant, one star)? Would having a restaurant in Córdoba with a green star (green stars indicate restaurants with environmental, resource management and waste disposal initiatives) attract tourists, considering that Córdoba does not currently have such restaurants? What types of gastronomic tourist does Córdoba receive? What are their main characteristics?

To answer these questions, oil, wine and ham PDOs in the province of Córdoba were analyzed, the demand profile and existing supply related to each gastronomic product was investigated, and a gastronomic tourist profile was identified for each product, in case different marketing campaigns are necessary for different tourist profiles.

To analyze the aforementioned, univariate and bivariate descriptive statistics were used to identify the degree of association between variables, and interviews were conducted with owners of restaurants and wineries, oil mills and ham curing facilities with the objective of identifying gastronomic tourist profiles. SARIMA models were applied to predict the future demand of gastronomic tourists for oil, wine and ham, providing a vision of the potential demand. The results obtained indicate that the demand for gastronomic tourism will grow, but not as quickly as rural tourism after the pandemic.

To improve the demand for gastronomic tourism, a series of strategies are proposed so that the growth in demand for gastronomic tourism in Córdoba will be higher than expected.

After this introduction, in the second section, designations of origin in Córdoba are analyzed. The third section contains a brief review of the existing scientific literature in this field. The fourth section presents the methodology used in this study. The fifth section presents the main results of the research. The last two sections provide a discussion and conclusions.

## 2. Protected Geographical Indications and Designation of Origins as Quality Hallmarks of Agri-Food Products

New trends in consumer habits have led to a growing interest in gastronomic products of higher quality that are differentiated and adapted to the new needs of different groups and market segments [8,14]. This increase in the consumption of differentiated products based on their quality is obtained through geographical indicators of origin and, in particular, PDOs, which integrate in their definition not only geographical origin but also the relevant forms, traditions and specialization involved when producing high-quality products, as well as production regulation and control mechanisms [15]. To increase their competitiveness and expand their market share, agri-food companies try to establish differentiation strategies for their products based on highlighting differences in attributes, materials or characteristics with respect to a competitor’s product.

In Spain, there are 199 agri-food product PDOs and 159 PGIs; therefore, Spain has a great variety of quality food products, especially wine (97 PDO), oil (30 PDO) and cheese (27), indicating that Spain is a good gastronomic destination (Table 1).

The province of Córdoba has seven PDOs, among which oil accounts for four, with one each for wine, Iberian ham and vinegar. The most visited is the wine PDO, with more than 30,000 tourists per year [17]. The PDOs are detailed below:

- Iberian Ham: “Los Pedroches” PDO. Located to the north of the province and encompassing 32 municipalities with an area of 3612 km^2^, the predominant climate in the area is Mediterranean but with a certain continental touch and winds from the Atlantic, with cold winters and long, dry and hot summers and with little rainfall. This climate, together with the structure of the soils, is favorable to the growth of oaks and their fruit (acorn), which is the sustenance of the pigs from which the ham is obtained), forming a *dehesas* (savannah-like open woodland), a typical ecosystem in the area.
- Wine: Montilla-Moriles Designation of Origin. This environment encompasses a total of 17 municipalities and more than 5000 hectares of vineyards, and distinguishes an area differentiated by the type of soil, considered “Superior” (approximately 1600 hectares). The main grape types are Pedro Ximénez, Layren, Baladí, Verdejo, Moscatel de Grano Menudo, Moscatel de Alejandría, Torrontés, Chardonnay, Sauvignon Blanc and Macabeo.
- Vinegar: Vinagre de Montilla-Moriles Designation of Origin. These vinegars are obtained exclusively by the acetification of PDO wines and have been produced with PDO wines since 2015.
- Oil: The province of Córdoba is the second largest nationwide in terms of olive oil production, with 317,000 tons of oil produced in the 2020–21 season [18]. It has four designations of origin:
  o Baena Designation of Origin: This was Spain’s first agri-food Designation of Origin (1971). It encompasses eight municipalities and covers an area of approximately 60,000 hectares, with an average annual production ranging from 30 to 45 million kilos of oil.
  o Priego de Córdoba Designation of Origin (1995): This Designation of Origin encompasses the municipalities of Almedinilla, Carcabuey, Fuente Tójar and Priego de Córdoba, covers approximately 30,000 hectares, and is located in the southwest of the province of Córdoba, in Sierra Subbética park.
  o Aceite de Lucena Designation of Origin: Located in the south of the province of Córdoba, it encompasses 10 municipalities; it is the newest of the four Designations of Origin (2008) and comprises 73,000 hectares.
  o Montoro—Adamuz Designation of Origin (2007): This Designation of Origin encompasses the municipalities of Montoro, Adamuz, Espiel, Hornachuelos, Obejo, Villaharta, Villanueva del Rey and Villaviciosa de Córdoba, as well as the northern part of the municipality of Córdoba. It contains approximately 55,000 hectares of controlled olive groves.

Three of these products (wine, olive oil and ham) can contribute to directly and indirectly increasing the wealth of the region through business activities such as tourism, but there is a need to raise awareness within the business sector to synergize production activity (agriculture/livestock) and services (tourism). The symbiosis of both activities will allow the creation of a tourism product that makes Córdoba more attractive as an international gastronomic destination.

**Figure 1:**
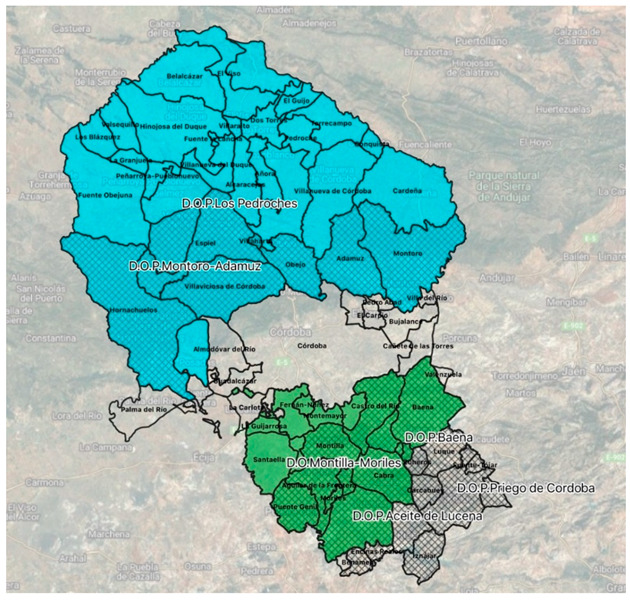
Geographical location of PDOs in the province of Córdoba. Source: own elaboration.

Figure 1 presents a map of the province of Córdoba, where practically all the municipalities of the province, except the capital, are under some designation of origin, with some included in more than one.

In the figure, blue represents the Pedroches ham PDO; green represents the Montilla-Moriles wine PDO, and the hatched area represents the four olive oil PDOs.

In recent years, there has been greater interest in Córdoban food products and tourism, materializing, on the one hand, with the expansion of restaurants linked to popular cuisine and local quality products endorsed by PDOs and PGIs, and, on the other hand, in the consolidation of the gastronomic tourism area [3,4,19,20,21], turning this type of tourism into an important dynamic element of the economy and culture of the areas where they are located.

The development of gastronomic tourism in the interior areas of Córdoba contributes to integrating the traditional primary productive function with the specialized tertiary industry, increasing the sources of income, improving the levels of income and employment for the local population, and generating multifunctionality in rural territories.

However, to commercialize gastronomic products from the tourist’s point of view, gastronomic routes must be created that include producers, restaurants, shops, etc.; that is, public entities and private companies that work on creating a gastronomic circuit (gastronomic routes), which can solve the difficulties of commercializing regional food products because these circuits are instruments to promote such foods. In this way, the use of geographical quality indicators makes it easy for consumers to recognize the superior and differentiating qualities of each product. Strengthening the origin or provenance attributes of products has thus become an important marketing tool for the commercialization of products and brands, especially if these brands belong to the agri-food sector [22]. The place of origin or provenance of products can become an important source of competitive advantage for companies, capable of influencing consumers when valuing products or brands [23].

Gastronomic routes are defined as itineraries that allow the recognition and enjoyment of the agricultural and industrial production process and the tasting of regional cuisine considered an expression of regional cultural identity. These routes consist of producers who welcome tourists in their establishments and provide them with food services and regional restaurants that showcase traditional dishes based on local primary production and agroindustries in the area. They are organized around a key product or, in some cases, around a basket of products that characterize the route and give it identity; such itineraries are developed using road networks [24].

There can be countless activities related to the products with which a route is identified: visits to producers, who receive tourists in their establishments, showing them the preparation process and allowing them to taste the products; visits to restaurants that offer traditional dishes with local products; and visits to museums that relate product and place, among other activities.

When designing a gastronomic route, public entities and producers should link tourism with food and at no point ignore other attractions that link the food and beverage cluster with tourism, as doing so usually leads to the loss of development and market opportunities for both.

Among the elements that characterize a gastronomic route are (a) the production that distinguishes it from another region, (b) the itinerary developed along a road network, (c) the establishments attached to the route that produce, distribute or advertise the food that highlights the route, (d) a minimum number of participants along each route that justifies its opening, (e) a regulatory norm that regulates the functioning of the participants, (f) a regional menu whose dishes have been prepared with the products that characterize the route, (g) a local organization, association or tourism office that offers information about the gastronomic route, (h) the signage for the route and a map that provides explanatory information about it, and (i) culinary offerings of the product in restaurants and establishments in the area [25].

**Figure 2:**
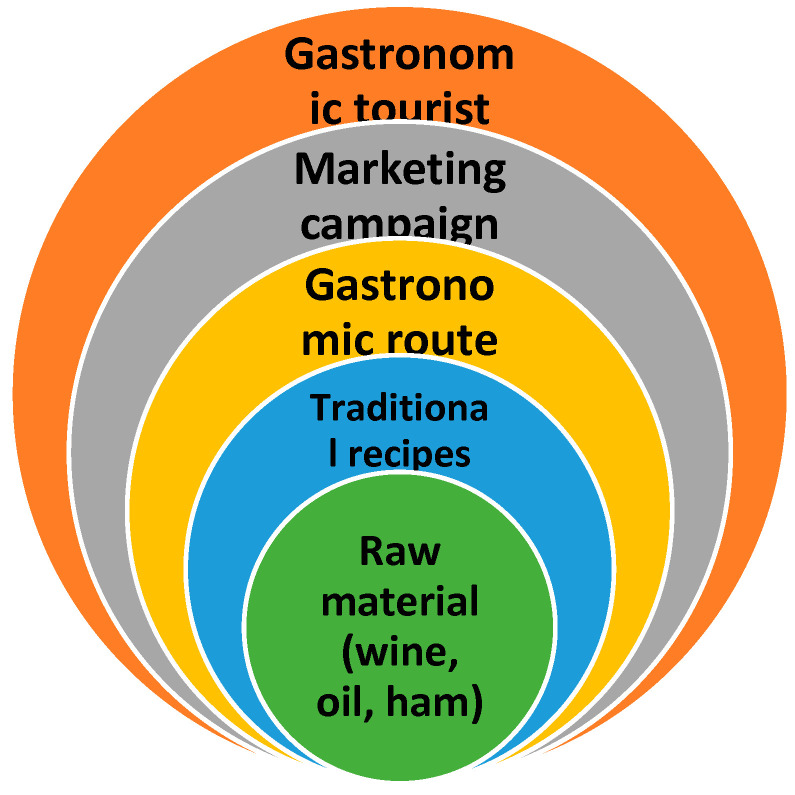
Pillars of gastronomic tourism in the province of Córdoba. Source: own elaboration.

For gastronomic tourism to be successful, it is necessary, first, to have a quality raw material and, second, to have good dishes prepared from those materials whose recipes are typical of the area, to have a good gastronomic route, which includes not only the places that produce the raw material (vineyards, dehesas, curing facilities, oil mills, wineries, etc.) but also restaurants where tourists can sample the traditional food or customs of the area, accommodation, shops, etc.., and to have a well-structured offering that is part of the gastronomic route. However, if a gastronomic route is not publicized or known, few tourists will visit it. To achieve this, marketing campaigns in specialized magazines, international fairs, etc., are needed to create awareness among potential gastronomic tourists so that they will visit Córdoba and be interested in its gastronomy. These actions will serve as the bases or pillars to attract gastronomic tourists to the gastronomic destination of Córdoba (Figure 2).

## 3. Literature Review

Gastronomic tourism is based on a combination of factors such as food, culture, the geography of the destination and the availability of infrastructure to support tourism. Studies have shown that the culture and environmental factors of a destination shape its gastronomic identity. While the geography and climate of a destination determine the type of food that can grow in the area, history and tradition influence the cuisine and eating habits of the destination and thus collectively determine its gastronomic identity [26].

There is a large amount of scientific literature that analyzes gastronomic tourism from different perspectives:

- Territory: Gastronomic tourism has been studied in specific regions or countries, such as France, in which Batat [27] investigated the role of Michelin-starred chefs as change-makers and advocates of tourism activities in both rural and urban areas; Italy, in which Privitera et al. [28] analyzed the opportunities of gastronomic tourism for local development around the Sicily region; Greece, in which Pavlidis & Markantonatou [29] analyzed the promotion of gastronomic tourism in the northern regions of Greece; Singapore, in which Chaney & Ryan [26] described the evolution of gastronomic tourism in Singapore; Malaysia, in which Sanip and Mustapha analized the sustainability of gastronomic tourism in Malaysia [30,31]; Mexico, in which Correa [32] determined the factors that can help restaurants to be more competitive in the face of a health crisis in the state of Zacatecas; and Kenya, in which Josphine [33] carried out a critical review of gastronomic tourism development in Kenya.
- Product: Gastronomic tourism has been investigated based on a specific product, such as cheese, in which Medeiros et al. [34] carried out a study on the artisanal cheese of Serro; wine [17]; olive oil, in which Dancausa et al. [3] analyzed olive oil as a gourmet ingredient in contemporary Andalusian cuisine; ham, in which Millán et al. [35] investigated ham tourism as an opportunity for development in rural areas; tea or coffee, in which Seyitoğlu & Alphan [36] examined the tea and coffee museum experience of travelers from around the world; fish, in which Pratiwi’ [37] study aims to develop gastronomic tourism based on fish on the island of Belitung; or prepared dishes [38].
- Motivation: Such studies investigate what motivates tourists to undertake gastronomic tourism [39]. Decrop [40] analyzes the concept of motivation by emphasizing four different components (motives, needs, desires and benefits). Other authors, such as Hernandez & Dancausa [38], distinguish between types of tourist who visit gastronomical destinations, finding that motivation can be a main or secondary factor and classifying gastronomic tourists as follows. Gastronomy connoisseurs (gourmet tourists) are well-versed in gastronomy, and their main motivation for travel is to taste different products or typical dishes of the destinations they visit, as well as to purchase said products and learn in situ. They usually travel continuously throughout the year visiting prestigious restaurants. Gastronomy enthusiasts do not have a high degree of education in gastronomy but know the world of gastronomy relatively well. They typically have a university education, and their main motivation for traveling is to experience firsthand what they have read in different specialized magazines. Individuals interested in gastronomy do not have technical training in gastronomy but are interested in the world of gastronomy. Their main motivation for traveling is to experience typical dishes or products of their destinations, although not exclusively but as a complement to other tourist activities. Gastronomy novices, for various reasons (such as in response to advertising or a desire for new experiences), visit restaurants, wineries, or oil mills despite lacking knowledge regarding gastronomy. Their main motivation for traveling is not related to gastronomy, but they secondarily dedicate a few hours of their journey to gastronomy.

Gastronomy fits well as the main motivation (for gastronomy connoisseurs and enthusiasts, who visit a place specifically to enjoy its culinary offerings) and as a secondary motivation (for example, for those for whom, although their main motivation is not to learn about the gastronomic richness of a destination, do consider gastronomy as a tourism option) for engaging in gastronomic tourism. In turn, as a main or secondary motivation, gastronomy can represent an intensification or extension of daily life. Whatever the motivation, such tourists can experience completely different gastronomy from that of their place of origin [41,42,43,44].

- Satisfaction: These studies analyze how satisfied tourists are with the gastronomy of the destination they visit [45,46,47], investigating how well a destination performs through an analysis of tourist satisfaction, with one of the most important factors when choosing a holiday destination being satisfaction with previous stays [48,49].
- Consumption: Studies investigate gastronomic tourism from the perspective of hedonistic theory, which stipulates that tourists will consume food for the sake of experiencing it and not for hunger, and thus, food consumption is more experiential than functional [50,51].
- Gastronomic tourist profile: These studies analyze gastronomic tourist profiles [52,53] using various techniques, such as neural networks [54], cluster analysis [55], factor analysis [35] and tourism demand forecasting models using ARIMA models [56,57,58].

Many studies on gastronomy investigate different factors, e.g., motivation, satisfaction, territory, product, etc., all of which are equally relevant, allowing the characterization of tourists to better understand their tastes and preferences, facilitating the construction of a more adequate tourism product adapted to the demand [59].

The novelty of this research is that it analyzes the entire gastronomic products associated with PDOs in Córdoba; existing studies focus on specific raw materials, not all materials, nor do they compare them. In addition, strategies are proposed to improve this tourist segment. The following two hypotheses are tested:

**H1:**  *The profile of gastronomic tourists depends on the product they consume*.

**H2:**  *Gastronomic tourism in the province of Córdoba will expand after the pandemic*.

## 4. Materials and Methods

The primary data sources of data were obtained through fieldwork that involved surveying a population of tourist consumers who visited one of the seven PDOs in the province of Córdoba in 2021–2022, with the objective of determining which factors influence gastronomic tourists. To this end, a questionnaire was administered to verify its validity as a measurement instrument according to [60,61], and a pre-test was performed with 40 gastronomic tourists to verify that it satisfied the following criteria:

1. Simple, viable and accepted by tourists and researchers (viability).
2. Reliable and accurate, that is, with error-free measurements.
3. Adequate for the problem to be measured.
4. Reflects the underlying theory in the phenomenon or concept to be measured (construct validity). Questionnaires similar to those used by previous gastronomic tourism researchers were used [3,4,13,17,62,63,64,65,66].

A questionnaire consisting of 38 items divided into four blocks (Table 2) was designed. The first block collected personal information (age, gender, level of education, marital status, etc.). The second block collected information about the gastronomic route taken (how the tourist found out about the gastronomic route, if the route met his/her expectations, what would improve the route, purpose for traveling the gastronomic route, etc.). The third block collected information on the motivation for gastronomic tourism (the reason for choosing the gastronomic route) and a self-classification of the tourist’s level of gastronomic tourism. The fourth assessment block (on the services received during the route, price of the trip, hospitality and treatment received, etc.) was a questionnaire directed at the population of tourist consumers visiting a gastronomic route/PDO in Córdoba. Access by the investigators to the routes (curers, oil mills, wineries, restaurants, etc.) and permission to conduct interviews with tourists was authorized by the managing body and owner of each PDO.

Prior to responding to the questionnaire, tourists were informed of the academic purposes of the study and the anonymity of their answers. Verbal consent was obtained prior to administering the questionnaire. At all times, the visitor’s anonymity was guaranteed.

With the information obtained in the survey, the following were carried out:

1. A univariate descriptive analysis was conducted to determine the profile of tourists, segmented by product, i.e., wine, oil and ham, with the objective of identifying if there are significant differences between tourists.
2. A bivariate analysis was conducted using contingency tables to identify whether there is an association or independence between two variables, using the χ2 statistic (where H_0_ is that the analyzed variables are independent and H_1_ is that the analyzed variables are related). The aim of said analysis was to determine the associations between variables, thus allowing the identification of the profiles of gastronomic tourists.
3. A SARIMA model (used in previous studies of tourists, such as those by Lim [67] to predict tourist demand in Macao after the COVID-19 pandemic; Yang [56] to predict tourist demand in 29 Chinese regions, and Petrevska [57] to predict tourist demand in Macedonia; Zhang [58] to predict tourist occupancy in a hotel) was used to predict the potential demand of gastronomic tourists in Córdoba, based on a sample (61 observations) collected from February 2015 to May 2022. ARIMA models, popularly known as the Box–Jenkins (BJ) methodology, analyze the probabilistic, or stochastic, properties of economic time series themselves [58]. In this case, this was the number of gastronomic tourists in Córdoba.

## 5. Results

Table 3 provides the characteristics of gastronomic tourists in Córdoba; the data have been categorized by product to determine specific profiles, with the objective of testing Hypothesis 1. The segmentation of the tourist profile by product indicates that there are a series of common variables, such as gender, because the majority of the gastronomic tourists were male, with the highest percentage in oil tourism (58.2%), had a high school/secondary education level (43.8% of ham tourists, 40.1% of enotourists and 38.5% of oil tourists), were married, travelled accompanied and came mainly from Andalusia (58.9% of ham tourists, 43.1% of enotourists and 59.4% of oil tourists). For place of origin, there was a greater than 15-point difference between some products, because enotourism, which is better known internationally, draws a greater number of foreigners than do ham or oil, which are not known as tourist products in international markets. More than 60% of tourists worked for others and used vacations to travel to Córdoba; travel was very seasonal, and they spent less than 24 h in the province of Córdoba. Many classified themselves as excursionists because they spent less than six hours at a destination. Therefore, they did not spend the night, which is a problem for the city. Overnight stays, with respect to the average daily expenditure, are between EUR 66 and 100 for ham tourism and oil tourism, and higher than EUR 100 for enotourism. The average income was between EUR 1500 and 2000 for olive oil tourists and ham tourists, and between EUR 1000–1500 for enotourists. This result is significantly different from those reported in other relevant studies of Andalusia [53,54,55,56,57,58,59,60,61,62,63,64,65,66,67,68,69], where enotourists who visited Andalusia had greater purchasing power (EUR 2500) than ham and oil tourists, because many enotourists are international tourists who visit the Xeres PDO, which receives more than half a million visitors per year, the vast majority of which are foreign tourists with high purchasing power. If similar studies on the profile of oil tourists are analyzed, such as that by Alonso and Northcote [70] in Australia, a similarity is observed in terms of with whom the trip is made, where few tourists make the trip alone. For Córdoba, 2% of oil tourists and 4.7% of enotourists travelled alone, compared with 2% reported in other studies of gastronomic tourism, such as that by Olivera [71]. In Mealhada, Portugal, some variables are similar, such as a higher percentage (56%) of men choosing this type of tourism as well as the level of education of tourists. In a study by Orgaz and Lopez [72] in the Dominican Republic and a study by Huertas et al. [48] in Mocha Canton, Ecuador, the tourists were mostly male (56% and 51%, respectively, in both countries), but the age distribution was different. In Córdoba, approximately 30% of gastronomic tourists were between 50 and 59 years of age (30.5%) with medium purchasing power; in contrast, in the study by Orgaz and Lopez, gastronomic tourists were younger (30 to 39 years old), and in the study by Huertas, such tourists were between 21 and 27 years old (27%), mainly with low purchasing power.

Regarding routes (Table 4), this type of tourism is popularized mainly through the internet or social networks (ham tourism, 61.4%; enotourism, 35.8%; and oil tourism, 48.1%). Because ham is a product that is not well known in international markets, social networks and the internet are the best disseminators of information about this tourist product. With respect to the expectations tourists had of routes, almost 80% reported that their expectations were met (ham tourism, 84.2%; oil tourism, 80.6%; and enotourism, 79.2%). The percentage for enotourism may be lower because many enotourists have already experienced other wine routes and draw comparisons, indicating that it is necessary to improve the Montilla-Moriles route, especially the explanation of the route (40.1%) and to improve the enotourism and oil tourism routes in general through better signage (50.6% and 47.7%, respectively). The degree of satisfaction with the three products exceeded 76% for more than 80% of the interviewees, with oil tourists being the most satisfied and those who would be willing to repeat the visit using a similar route (96.6%). This product, unlike wine, can only be found in countries of the Mediterranean basin, where there is a good climate and other tourist activities can be carried out.

The main motivation for gastronomic tourism (Table 5) was to learn the process of making oil (50.3%), wine (65.8%) and ham (50.5%), with wineries, ham curing and oil mills being the main attractions. At the end of the visit, tourists have the opportunity to taste the product and better appreciate the quality of the wines, hams and oils in the area. Among the respondents, 97% agreed with the creation of a combined route of gastronomic products, because they thought that the route would be gastronomically enriched and would be more attractive through the pairing of products, e.g., oil–ham and oil–cheese (Table 5).

The results of Table 3, Table 4 and Table 5 indicate that Hypothesis 1 was not fulfilled (the profile of gastronomic tourists depends on the product they consume) due to the similarity in the classification of the variables. In Table 3 (personal characteristics of gastronomic tourists) where 10 variables were analyzed, seven of them had the highest classification in the same category, coinciding in 70% (age, education level, gender, who the tourist travelled with, where the tourist was from, employment situation, and duration of the trip). In Table 4 (questions about the visit) where nine variables were analyzed, seven had a similar classification, coinciding in 77.7% (number of people who travelled the route with the tourist; whether the PDO or the gastronomic route met the tourist’s expectations; whether the tourist would be interested in receiving more information after the visit; whether the price paid seemed to be consistent with the route; whether the tourist came expressly for this gastronomic route, or if it was offered to them in Andalusia; whether the tourist would repeat the experience using a similar route; degree of satisfaction with the visit), and in Table 5 (motivations) of the three variables analyzed, two of them had the maximum similarity of 66.6% (What were the reasons for the visit?; What would you think about the creation of a combined route of various gastronomic products with theatrical performances?). Therefore, the profile of the gastronomic tourist is similar in terms of ham, olive oil and wine.

**Figure 3:**
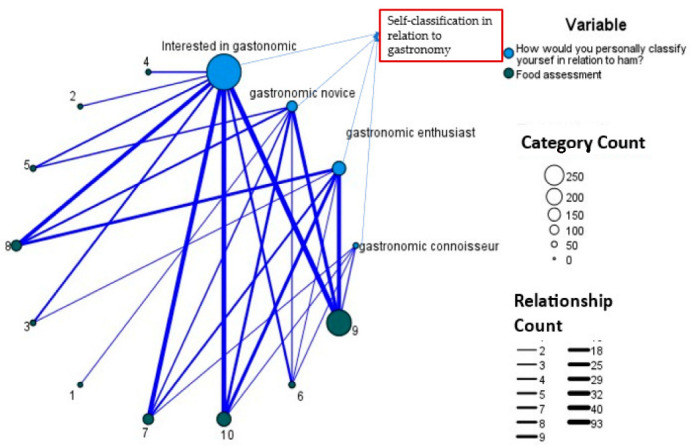
Relationship map. Source: own elaboration.

Figure 3 shows a map of relationships between gastronomic tourist classifications and scores given to gastronomy in Córdoba. The blue circles represent the personal classification variable with respect to gastronomy. The majority of tourists classified themselves as people interested in gastronomy (largest blue circle). They did not have training in gastronomy. Although one of the motivations for traveling to Córdoba is gastronomy, it is not the main reason. These tourists rated gastronomy favorably, with thick blue lines representing the highest scores (7, 8, 9, 10). Gastronomy connoisseurs (gourmet tourists) were the minority (smallest blue circle). They have vast gastronomic training and come to Córdoba expressly for its gastronomy. This type of tourist rated Córdoba food very positively, with scores ranging from 6 to 10 for gastronomy in Córdoba. For tourists who classified themselves as gastronomic novices, their expectations were met the least with respect to Córdoba’s food, in part because they did not understand gastronomy and had not visited other gastronomic destinations to appreciate the differences. However, on average, the gastronomy in Córdoba was very well rated, with the largest green circles representing scores of 9 and 10, indicating that Córdoba is a good gastronomic destination; however, this resource is not sufficiently exploited.

For a more in-depth analysis of the relationship between the different variables, bivariate analysis was carried out (Table 6). There is a strong relationship between tourist age and motivation to engage in gastronomic tourism (χ2 = 325.71, *p* ≤ 0.001): the older the tourist, the more positively he/she valued gastronomic tourism. Age also influences knowledge of gastronomy (χ2 = 62.32): younger tourists’ main motivation was to visit oil mills, wineries, ham curing facilities and learn about the production process, and older tourists’ (aged over 50 years) main motivation was tasting dishes that feature olive oil, wine or Iberian ham. Additionally, gender was related to satisfaction (χ2 = 11.88), with women giving lower scores to the degree of satisfaction. Tourists who use oil, wine or ham more often (daily) rated such gastronomic products more positively because they appreciate the raw material more when they use it every day (χ2 = 62.32). Younger tourists used new technologies, the internet, social networks and tourism websites to learn about tours, while older tourists engaged in tours based on recommendations from friends and family. There is also a strong relationship between the reason for engaging in gastronomic tourism and age.

In addition, education level and degree of satisfaction are related: tourists with a higher education level had a somewhat lower degree of satisfaction, either because they believed the explanations about the production process were not very accurate or because they believed that there was a need for more audiovisual media (χ2 = 264.89).

**Figure 4:**
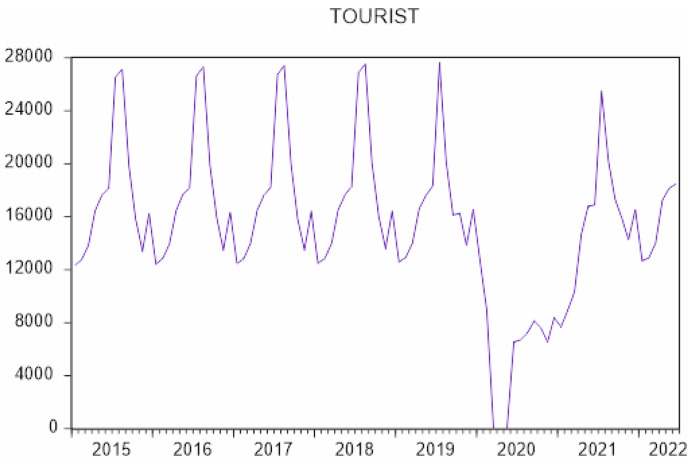
Monthly evolution of the number of gastronomic tourists in Córdoba (January 2015 to June 2022). Source: own elaboration based on information from the Designations of Origin of Córdoba. Vertical axis is thousands of people.

To forecast the demand for gastronomic tourism in Córdoba, ARIMA models were used in an attempt to quantify the evolution of this type of tourism from January 2015 to June 2022 (Figure 4) and to determine the effect of the COVID-19 pandemic on this tourism segment, with the aim of testing Hypothesis 2.

There are few studies that attempt to forecast the demand of gastronomic tourism, hence the importance of this study. To predict this demand, we collected monthly information from January 2015 to July 2022 on the number of gastronomic tourists who visited PDOs/gastronomic festivals in Andalusia. To model the number of gastronomic tourists (tourist), the BJ methodology was used to design a seasonal ARIMA (SARIMA) model (Table 7 and Table 8), where a variable is analyzed with reference to its past values.

Φ(B) ϕ(B) (1 − B)^d^ (1 − B^s^)^D^ Y_t_^(λ^ = ϴ(B) θ(B) a_t_

The demand for gastronomic tourism in Córdoba, called tourist in the model, is a variable with a nonnormal distribution, which has been corrected with Box–Cox transformation λ = 0.2 (tourist1 = tourist^0.2), and the mean trend was corrected with two differentiations in mean and 1 seasonal differentiation. This model is estimated to forecast the monthly demand for gastronomic tourism in Córdoba.

(1 + 0.497661B) (1 − B)^2^ (1 − B^12^) Tourist^0.2^ = (1 + 0.942206B^12^) a_t_

**Figure 5:**
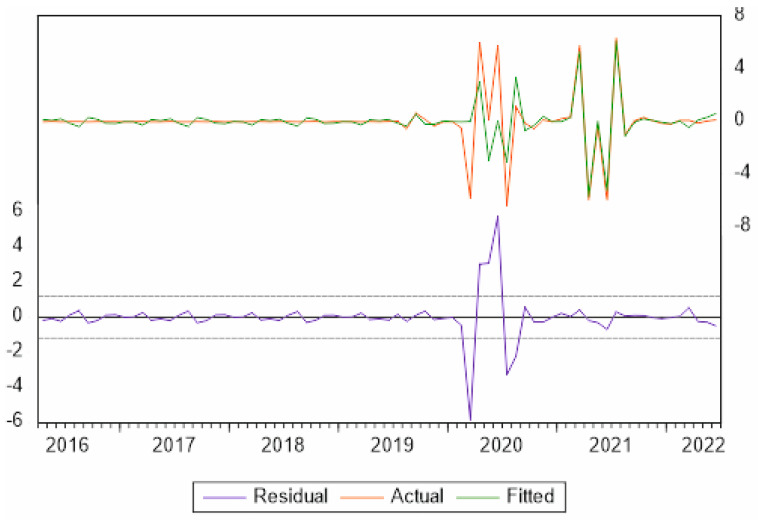
Monthly evolution of the number of gastronomic tourists in Córdoba and estimation errors (January 2015 to June 2022). Source: own elaboration based on ARIMA model estimate.

Figure 5 shows the evolution of the gastronomic tourist variable (current), the values estimated by the model for those same dates (fitted) and the errors committed by the estimated model (residual), observing that these residuals are normal when between the range of ±2 Sd (standard deviation), except for 2020, when there were months that there was no tourism due to travel restrictions, resulting in abnormal estimates.

This model was used to forecast the demand for gastronomic tourism after the pandemic, which indicates that the expected growth will cause this type of tourism to exceed pre-pandemic levels. This may be because tourists seek natural environments as destinations, considering them safer. In fact, this type of tourism is more individualized and usually occurs in small groups; however, it is still considered seasonal tourism [73], distinguishing itself from gastronomic tourists who usually visit the provincial capital in the months from May to September. These tourists want to taste dishes made with quality products from the area; in contrast, gastronomic tourists who visit the province tend to concentrate their travel around the olive harvest (October–January), grape harvest (September and October), and pig slaughter (November–December), when tourists can participate in olive pole-beating (hitting branches to make the olives fall), grape harvesting, or the preparation of pork meat products after slaughter. Thus, although olive groves, vineyards, dehesas, oil mills, wineries and ham curing facilities, as well as olive, wine and ham museums, can be visited throughout the year, demand continues to be seasonal.

Table 9 provides a comparison of the real data for the number of tourists before and after the pandemic in Córdoba. The predictions for the months of July to December 2022 indicate an increase of 13,982 gastronomic tourists compared to the numbers before the pandemic (year 2019), indicating a recovery of this sector with an increase of 12% higher than other types of tourism. This finding suggests that after the pandemic, tourists have preferred to visit inland environments related to nature and seek quality gastronomic products; however, although these figures are positive, the number of gastronomic tourists is still low compared to other Spanish and international gastronomic destinations.

From the previous predictions, it can be affirmed that gastronomic tourism is a market niche still to be sufficiently exploited in Córdoba, especially oil tourism and ham tourism, having great potential for development in the province (growth greater than 12%). Therefore, Hypothesis 2 is confirmed, indicating that gastronomic tourism in the province of Córdoba will expand after the pandemic, but that this growth could be greater with adequate promotion given the uniqueness of these gastronomic products, which cannot be found in other places in the world, especially Iberian ham, making Córdoba a special destination.

## 6. Discussion

Based on research on gastronomic tourism and the results of this study carried out in the province of Córdoba, the profile of tourists can be different; for example, of those who attend the Trujillo Cheese Festival and the Cherry Blossom Festival in the Jerte Valley in Extremadura (Spain), the main tourists are women, 54.4% [74]; however, in the province of Córdoba, men predominate as tourists for all products studied (57.1% ham tourists, 51.2% wine tourists and 58.2% oil tourists), with the percentage of women being lower (42.5% ham tourists, 49.8% wine tourists and 41.8% oil tourists). The education level among tourists is also different; in Extremadura, university students were the predominate respondents (49.5%), and in the study by Mejía et al. [75], 59% of the respondents who engage in gastronomic tourism were university students. In the province of Córdoba, those with a baccalaureate level of education account for 43.8% of ham tourists and 38.5% of oil tourists, with the highest level being almost 20 points lower in Córdoba. However, there is similarity with respect to the origin of tourists; 27.5% are from the Extremadura region, and 24.7% from a nearby region. In Córdoba, more than 43% are from Andalusia, reaching 59.4% for oil tourists. Therefore, gastronomic tourism does not require spending the night, unlike other tourist activities, especially beaches, where gastronomic tourists are mainly foreigners [72]. There is much similarity in the tourist profiles of the three products analyzed. Compared with that for other places or products, the degree of satisfaction exceeds 76% for more than 90% of the respondents, reaching 95.6%, indicating that the tourism products offered based on gastronomy are high quality, similar to the results obtained in the study by Clemente et al. [76] with respect to the profile of gastronomic tourists in Valencia, where 97% of those surveyed considered gastronomy in the area to be good or very good.

Gastronomic tourists in Córdoba are therefore satisfied and would repeat experiences, a finding that is similar to the results reported by Huertas [52] in Canton-Mocha (Ecuador), where more than 91% of tourists would return to that same gastronomic destination. In Córdoba, the high returns for ham tourism (82.3%) and oil tourism (96.6%) are due to the relationship between satisfaction and repeated experience.

**Figure 6:**
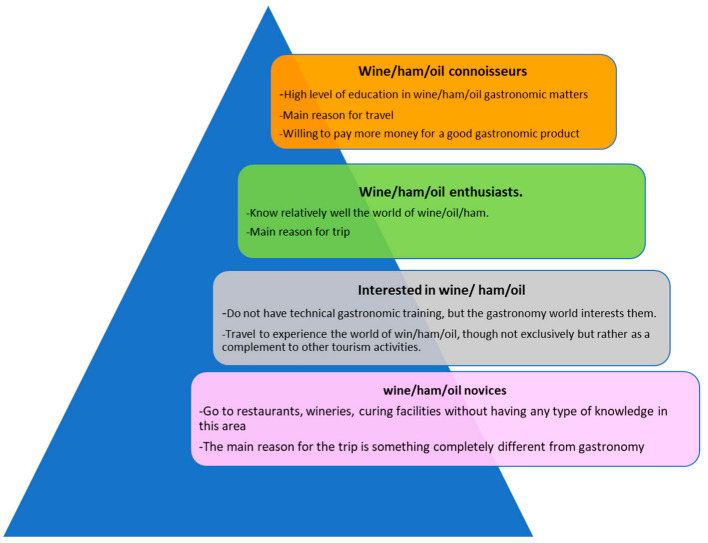
Classification of the gastronomic tourist according to product. Source: own elaboration.

In an analysis of products and tourists’ personal classification in relation to the products, Córdoba enotourists have an income of EUR 1001 to 1500, which is very different from the tourists who visit the Quinta da Gaivosa wineries in Portugal [77]. These enotourists can be classified in the highest category (Figure 6), wine connoisseurs, indicating that they are great connoisseurs of wine culture, travel expressly for reasons related to wine and earn more than EUR 3000 per month, buying wine in the winery after the visit. This is the type of tourist profile that the Montilla-Moriles route should seek.

Therefore, the tourists at the top of the pyramid (Figure 6) are the most selective and demanding, but those who travel to a destination know what product they are looking for, are willing to pay more for the gastronomic product and, if the experience is satisfactory, will repeat it, creating loyalty to that destination. A gastronomic destination has a life cycle similar to any tourism product. The basic concept of the life cycle in tourism studies, attributed to Butler [78], refers to the fact that tourist destinations show an evolutionary path formed by different stages: exploration, participation, development, consolidation, stagnation and decline or rejuvenation; for example, within gastronomic tourism destinations, there are some that are emerging, such as oil tourism [79], or are very developed, such as the wine routes in the Napa Valley [80]. To transition Córdoba into a gastronomy reference, tourists who have been introduced to the product must become more familiar with the product, and their advertising of the destination will encourage other tourists to come in the future, with the aim of becoming an exclusive gastronomic destination for gastronomy connoisseurs. Given the quality of the products found in the province of Córdoba, good tourism planning and marketing could turn it into a gastronomic destination similar to France, as indicated by Cunha [81]. Increasingly, there is a need to focus on promoting the values of intangible heritage, such as the gastronomy of a region, both for residents and tourists. Due to interactions with other cultures, gastronomy should always be understood as part of the cultural experience of a country or region. Gastronomic richness must be supported by the competent local, regional and national authorities so that traditions and culinary values are not lost over time. Considering the importance of certified products for the gastronomic identity of a region, it is necessary to combat the extinction of several gastronomic traditions that may lack product certification and/or support from competent authorities. To increase the number of tourists, gastronomic routes should be created, similar to those designed in the Cunha study in the Dão-Lafões region (Portugal), which would encourage the number of overnight stays in Córdoba and would increase the richness of that area.

## 7. Conclusions

The province of Córdoba has three key materials from a gastronomic point of view, i.e., olive oil, which is part of the Mediterranean diet and present in all Andalusian cuisine; ham, as not only a nutritional element rich in protein, but also part of typical dishes such as *flamenquín;* and wine, as a drink that complements a dish and as a cooking element in typical recipes such as sirloin steak or meat with Pedro Ximénez wine, making Córdoba a high-quality gastronomy region. However, this culinary wealth is not being sufficiently exploited from the tourist’s point of view. A brand that identifies Córdoba as a gastronomic destination should be created; this approach will increase the number of tourists, because although tourism will grow by 12%, as determined using an ARIMA model, such growth is lower than that seen in other areas of the interior of the community [82] that have expanded by 147% after the pandemic. It is necessary to develop activities that increase the average expenditure of tourists and increase overnight stays in the city and province. For these two elements, average spending and overnight stays are among the lowest in Andalusia (EUR 68.6 and 2.9 days in Córdoba compared to EUR 72 and 5.1 days in Andalusia) [83].

The differentiated quality of the food products, the cultural tradition and their artisanal preparation make these products tourist attractions, especially considering the changes in tourist consumers’ habits (vacations more distributed throughout the year, although they are shorter, weekend trips, the desire for a healthy life, natural landscapes—the current situation, i.e., the pandemic, has contributed to this—environmentalism, etc.). The areas that host these PDOs have seen their wealth increased, and the quality of life of their populations has improved.

Finding tourists with a certain socioeconomic profile requires time. Tourists who have a high income level and a university level of education, want to spend the night in the area, and use the services that are available should be sought; however, for this to occur, these areas must be provided with those services and facilities (hospitality, restaurants, activities, etc.).

There are detractors of tourist activity. Such individuals think that tourism in the province of Córdoba will lead to the destruction of the rural environment they visit as a result of the explosion of services exceeding the carrying capacity of destinations, causing a loss of identity in the area and increasing housing prices, etc., which, although they could occur, are easily avoidable with proper planning.

The availability of unique foods and other gastronomic offerings at destinations must be complemented with a specific strategy for the promotion of gastronomic tourism for it to be successfully developed [33]. Based on our analysis of the three products, Córdoba has a greater potential for attracting ham and oil tourists because wine tourism already has well-established routes, such as those for La Rioja [84], and it will be very difficult to compete with them in the short and medium terms. Therefore, effort should be focused on the development and improvement of ham and oil routes.

Regarding the hypotheses, Hypothesis 2, which refers to growth of the demand for gastronomic tourism, is confirmed, but not at the desired levels, and Hypothesis 1 is not confirmed, because tourist profiles do not differ with respect to the gastronomic products they consume. This is due in part to the fact that tourists are from nearby and their personal characteristics and motivations are very similar.

Based on the obtained results, the following strategies are proposed (using the reference products wine, olive oil and Iberian ham) that could contribute to improving this tourism segment:

- Create plans and/or complementary activities that will encourage overnight stays in the area. These activities can range from festivities, samplings and fairs to tastings, training courses, competitions and stargazing, taking advantage of the low light at night in many of these rural areas, and lasting more than one day to encourage overnight stays.
- The above would make sense as long as a hospitality and catering infrastructure with the capacity to accommodate these types of events is adequately developed. For this, there must be investments in the area as a complement to the production of goods that is already being carried.
- This symbiosis between products with a designation of origin and geographical area, accompanied by corresponding marketing campaigns, would not take long to bear fruit [85].
- Develop signage within the area that encompasses the three products. Adequate signage, as part of street furniture in these rural areas, would contribute, both as publicity (for those who, at that time, are not engaging in tourist activities) and as information for tourists who wish to move from one place to another. Not surprisingly, through our research, lack of signage was identified as an item that need to be improved for designations of origin in the province of Córdoba.
- The seven designations of origin should combine their efforts not only to develop economies of scale that minimize the associated costs of advertising and promotion, but also, as all products are highly recommended both from the gastronomic and nutritional perspectives, to organize routes that cover one, two or all three products. For example, dinners or lunches could be organized between olive producers or vineyards, focusing on products of the land, after a day in which the tourists have visited an oil mill, a ham curing facility and/or a winery. Such events could be accompanied by live performances, if desired.
- Generate awareness of “sharing tourism” among the different designations of origin. That is, to offer a wider variety of activities and products so that potential tourists do not need to leave the area.
- Create an association among the seven PDOs in the province of Córdoba that would support their interests, with campaigns aimed at attracting investments and promoting tourism. The similarity of offerings and the areas in which they are developed make this type of union very viable because conflicting interests are highly unusual.
- All of the above would be much easier with the involvement and cooperation of economic agents and public administration. The management and processing of grants and subsidies, national and international promotion, public events, and the designation of protected areas or natural parks, etc., will be more effective if the highest possible levels of decision-making capacity participate.
- The implementation of these “recommendations” should be made with the supervision of a panel of experts related to different areas of knowledge, for example, economists, lawyers, agronomists, forestry engineers, architects, biologists, landscapers, etc., to minimize the risks that can lead to overcrowding natural landscapes of incalculable beauty as much as possible.

Good management of the gastronomic resources in Córdoba could make this province a well-known destination and valued at the national and international levels.

In conclusion, an increasing number of tourists seek specific learning experiences in which gastronomy plays a predominant and central role [86]. For Córdoba to be an internationally recognized gastronomic destination, it is necessary not only to have good raw materials and exquisite dishes but also to be recognized in international markets. This work serves as a barometer of the situation of this segment, aiming to help public agencies and private companies join forces and address the errors detected. The synergy among all actors will make Córdoba a high-quality gastronomic destination.

One limitation of this study was that it used a joint survey of tourists to the capital and to the province of Córdoba. The survey yielded an average gastronomic tourist profile for each product (wine, olive oil and ham). It would be interesting to differentiate gastronomic tourists visiting the capital from those visiting the province in case there are significant differences.

As future lines of research, it would be interesting to compare profiles of gastronomic products and differentiate between national and foreign tourists to achieve a better segmentation and to compare the gastronomic tourists of Córdoba with those of other World Heritage cities.

## Figures and Tables

**Table 1:** Distribution of agri-food products, wines and spirits with PDOs and PGIs in Spain, Andalusia and Córdoba (July 2022).

Agri-Food Products	PDO				PGI	
	Spain	Andalusia	Córdoba	Spain	Andalusia	Córdoba
Fresh meat (and offal)	-	-	-	22	1	-
Meat products	5	2	1	11	2	-
Cheese	27	-	-	2	-	-
Other animal products (honey)	3	1	-	4	-	-
Oils and fats (31 oils and 2 butters)	30	12	4	3	-	-
Fruits, vegetables and fresh and transformed cereals	26	3	-	36	3	-
Fish, seafood and fresh crustaceans and derived products	1	-	-	4	4	-
Other products (saffron, paprika, tiger nut, hazelnut, vinegar and cider)	9	3	1	-	-	-
Bakery, confectionary, pastry and dessert products	-	-	-	16	4	-
Cochineal	1	-	-	-	-	-
Total PDO and PGI of agri-food products	102	21	6	98	14	0
Wines with designation of origin (DO)	74	7	1	-	-	-
Wines with qualified design of origin (DO Ca)	2	-	-	-	-	-
Quality wines with geographical indication (QW)	10	1	-	-	-	-
Paid wines (PV)	11	-	-	-	-	-
Wines with geographical indication (GI)	-	-	-	41	16	2
Aromatized wines	-	-	-	1	1	-
Total PDO and PGI of Wines	97	8	1	42	17	2
Spirits with PGI	-	-	-	19	1	-
Total PDO and PGI	199	30	7	159	32	0

Source: Prepared by the authors based on information from the Ministry of Agriculture, Food and Environment and the European Commission, Directorate General of Agriculture and Rural Development [16].

**Table 2:** Technical aspects of the survey.

	Demand Survey
Population	Tourists of both sexes over 18 years old who visited a gastronomic route or PDO in Córdoba
Sample size	315
Sampling error	±4.2%
Confidence level	95%; *p* = q = 0.5
Sampling system	Simple random
Date of fieldwork	September 2021–January 2022

**Table 3:** Profile of gastronomic tourists in Córdoba (%).

Block	Question	Classification	Percentage of Ham Tourists	Percentage of Enotourists	Percentage of Oil Tourists
Personal characteristics of gastronomic tourists	Age	18–29 years	14.2	23.0	14.4
		30–39 years	27.1	28.4	27.3
		40–49 years	20.3	**32.0**	19.3
		50–59 years	**31.1**	13.2	**30.4**
		Over 60 years	7.3	3.4	8.6
	Education level	No completed studies	9.3	1.0	9.4
		Primary and secondary studies	19.6	32.9	18.6
		**Secondary Bachelor**	**43.8**	**40.1**	**38.5**
		Higher studies	27.4	26.0	33.5
	Gender	**Male**	**57.5**	**51.2**	**58.2**
		Female	42.5	49.8	41.8
	Marital status	Single	26.9	35.0	26.9
		**Married**	**47.7**	**44.0**	**48.2**
		Divorced/separated	25.2	20.3	24.3
		Other	0.2	0.7	0.6
	Monthly income level of the family unit	Less than EUR 1000	19.8	23.9	19.8
		EUR 1001–1500	19.8	**47.6**	20.0
		EUR 1501–2000	**30.3**	18.3	**30.0**
		EUR 2001–2500	20.0	7.4	20.0
		More than EUR 2500	10	2.8	10.2
	Who do you travel with?	Alone	3.2	4.7	2.0
		**Accompanied by my partner**	**48.9**	**45.6**	**49.4**
		With friends	37.7	32.3	38.4
		With relatives	10.3	17.4	10.2
	Where are you from?	**Andalusia**	**58.9**	**43.1**	**59.4**
		Rest of Spain (except Andalusia)	30.1	34.3	29.8
		European Union (except Spain)	10.0	12.5	10.2
		United States	0.2	0.1	0.2
		Rest of the world (except European Union)	0.7	10	0.5
	Employment situation	**Employed by others**	**61.4**	**70.1**	**62.2**
		Self-employed	10.2	8.5	10.2
		Retired	17.4	7.4	17.3
		Unemployed	8.3	3.4	8.1
		Student	2.7	10.6	2.2
	Duration of the trip	**Less than 24 h**	**53.7**	**83.2**	**54.2**
		1–3 days	34.3	9.7	33.3
		More than 3 days	12.0	7.1	12.5
	Daily expenditure	Less than EUR 30	11.2	11.9	11.1
		EUR 30–65EUR 66–100	24.0	17.6	23.9
			**42.1**	22.4	**43.9**
		More than EUR 100	22.7	**48.1**	21.1

Source: own elaboration. Bold indicates the highest value classification.

**Table 4:** Univariate results of the survey of gastronomic tourism on Andalusia: questions about the visit.

Block	Question	Classification	Percentage of Ham Tourists	Percentage of Enotourists	Percentage of Oil Tourists
Questions about the visit	Number of people who travelled the route with you	1 person	8.3	15.6	12.2
		**2 to 4 people**	**76.2**	**70.1**	**66.3**
		More than 4 people	15.5	14.3	21.6
	Has the PDO or the gastronomic route met your expectations?	**Yes**	**84.2**	**79.2**	**80.6**
		No	15.8	20.8	19.4
	What would you improve?	Nothing	0.3	1.3	0.9
		**Signage**	**50.6**	38.5	**47.7**
		Explanation of the route or the PDO	24.8	**40.1**	39.1
		More audiovisual media	18.3	15.9	11.7
		Other	6	4.2	0.6
	Would you be interested in receiving more information after the visit?	**Yes, if it is free**	**62.3**	**74.3**	**59.7**
		Yes, in any case	17.5	6.2	20.2
		I do not think it’s necessary	20.2	19.5	10.2
	Did you come expressly for this gastronomic route, or was it offered to you in Andalusia?	**I came expressly from my place of origin**	**76.1**	**59.7**	**49.4**
		It was circumstantial; they offered it to me	23.9	40.3	51.6
	Does the price paid seem to be consistent with the route?	**Yes**	**92.7**	**90.1**	**96.3**
		No	3.8	9.9	3.8
	How did you learn about the route?	Travel agencies	5.2	14.6	13.9
		**Online, through social networks**	**61.4**	35.8	**48.1**
		On the recommendation of friends and family	31.3	**45.8**	32.2
		Other media	2.1	3.9	5.8
	I would repeat the experience using a similar route	**Yes**	**82.3**	**87.1**	**96.6**
		No	17.7	12.8	3.4
	Degree of satisfaction with the visit	Less than 25%	6.0	4.4	0.6
		25–50%	4.2	7.9	1.1
		51–75%	8.3	5.4	2.7
		**76–99%**	**61.4**	**64.2**	**57.0**
		100%	20.1	18.1	38.6

Source: own elaboration Bold indicates the highest value classification.

**Table 5:** Univariate results of the survey of gastronomic tourists: motivations.

Block	Question	Classification	Percentage of Ham Tourists	Percentage of Enotourists	Percentage of Oil Tourists
Questions about the motivation for the visit	What were the reasons for the visit?	Learn the culinary tradition of the destination	40.2	31	39.6
		Learn the process of making oil/wine/ham and visit oil mills, ham curing facilities, wineries	**50.5**	**65.8**	**50.3**
		Attend gastronomic festivals	9.3	3.2	10.1
	How do you rate the current situation in terms of tourism management at sites like the ones you have visited?	Good	**49.3**	39.4	**51.6**
		Fine	29.1	**52.8**	27.9
		Bad	21.6	7.8	20.5
	What would you think about the creation of a combined route of various gastronomic products with theatrical performances?	I agree	**98.1**	**99.3**	**96.5**
		I do not agree; I prefer to visit a single gastronomic route and not several	1.9	0.7	3.5

Source: own elaboration. Bold indicates the highest value classification

**Table 6:** Bivariate analysis.

Associated Variables	χ^2^	df	*p*-Value
Age/Motivation for engaging in gastronomic tourism	325.71	8	<0.001
Satisfaction with visit/gender	11.86	4	0.019
Satisfaction/self-classification regarding gastronomy	62.32	16	<0.001
Satisfaction/Place of origin	241.47	16	<0.001
Satisfaction/Education level	264.89	12	<0.001

χ^2^ Chi-square statistic. Related variables, α = 0.05; df = degrees of freedom.

**Table 7:** Estimation of the SARIMA model.

Variable	Coefficient	Std. Error	t-Statistic	Prob.
AR (1)	−0.497661	0.101278	−4.913816	0.0000
SMA (12)	−0.942206	0.062879	−14.98443	0.0000

Significant parameters α = 0.05.

**Table 8:** GARCH test.

Variable	Coefficient	Std. Error	z-Statistic	Prob.
	Variance Equation			
C	1.14 × 10^−4^	2.47 × 10^−5^	4.620412	0.0000
RESID (−1)^2	13.83276	0.962786	14.36743	0.0000
GARCH (−1)	0.114353	0.012500	9.147971	0.0000

Absence of autoregressive conditional heteroscedasticity. Dependent variable: TOURIST. 1. Method: ML—ARCH (Marquardt)—Normal distribution. GARCH = C(1) + C(2) * RESID(−1)^2 + C(3) * GARCH(−1).

**Table 9:** Differences in tourist numbers before and after the COVID-19 pandemic in Córdoba.

Year/Month	Tourists	Year/Month	Tourists	Difference	% Variation
2022/07	28,110	2019/07	27,590	520	1.88
2022/08	22,345	2019/08	20,140	2205	10.94
2022/09	17,524	2019/09	16,130	1394	8.64
2022/10	18,132	2019/10	16,240	1892	11.65
2022/11	15,625	2019/11	13,830	1795	12.97
2022/12	22,736	2019/12	16,560	6176	37.29
total	124,472		110,490	13,982	12.65

Source: by authors. Column %Variation = tourists year 2022/tourists year 2019.

## Data Availability

The data presented in this study are available on request from the corresponding author.
